# Chemical Nose-Based Non-Invasive Detection of Breast Cancer Using Exhaled Breath †

**DOI:** 10.3390/s25072210

**Published:** 2025-03-31

**Authors:** Yosef Matana, Shai Libson, Barak Amihood, Zvi Boger, David Lieberman, Offer Zeiri, Yehuda Zeiri

**Affiliations:** 1 Biomedical Engineering, Ben-Gurion University of the Negev, Beersheba 8410501, Israel; matanay@bgu.ac.il (Y.M.); zviboger@gmail.com (Z.B.); 2 Breast Health Center Soroka Medical Center, Ben-Gurion University, Beersheba 8410501, Israel; shaili@clalit.org.il; 3 OPTIMAL—Industrial Neural Systems, Beersheba 84243, Israel; barakamihood@gmail.com; 4 Pulmonary Unit, Soroka University Medical Center and the Faculty of Health Sciences, Ben-Gurion University of the Negev, Beersheba 8410501, Israel; lieberma@bgu.ac.il; 5 Department of Analytical Chemistry, Nuclear Research Center Negev, Be’er-Sheva 84190, Israel

**Keywords:** exhaled breath, breast cancer, data analysis, machine learning

## Abstract

**Highlights:**

**What are the main findings?**

- Exhaled breath was analyzed using a commercial electronic nose for early breast cancer detection.
- Samples were collected with no prior requirements from the patients.
- After feature extraction, the processed data was used for model optimization and training.

**What is the implication of the main finding?**

- Accuracy, precision, and specificity of 91.0% were achieved by the model.
- The method could provide a path for a rapid, simple, screening technique for low-income countries.

**Abstract:**

Breast cancer (BC) is the most commonly occurring cancer in women and one of the leading causes of cancer death in women worldwide. BC mortality is related to early tumor detection, highlighting the importance of early detection methods. This work aims to develop a robust, accurate and highly reliable, non-invasive, low-cost screening method for early detection of BC in routine screening using exhaled breath (EB) analysis. For this, exhaled breath samples were collected from 267 women: 131 breast cancer patients and 136 healthy women. After collection, the samples were measured using a commercially available electronic nose. The signals obtained for each sample were first processed and then went through a feature extraction step. An SVM model was then optimized with respect to the accuracy matrix using a validation set by applying a Monte Carlo cross-validation with 100 iterations, with each iteration containing 20% of the data. The validation set results were 80, 94, 88, and 95% for recall, precision, accuracy, and specificity, correspondingly. Once model optimization had concluded, 22 unknown samples were analyzed by the model, and an accuracy, precision, and specificity of 91% was achieved.

## 1. Introduction

Breast cancer (BC) is a leading cause of death in women, with 670,000 global deaths by BC in 2022 alone [1,2]. Although BC is sometimes thought of as a disease of the developed world [3], about half of BC cases and approximately 60% of BC deaths occur in less-developed countries [4]. Furthermore, BC incidence is increasing in developing countries due to increased life expectancy and changes in lifestyle [5]. In the United States, BC is the most commonly diagnosed cancer in women and the second most common cause of cancer-related mortality among women. It is estimated that over 310,000 women will be diagnosed with invasive BC in 2024, and it was estimated that approximately 42,000 will die from the disease [6]. Feminine gender and advancing age are the most significant risk factors for BC; hence, efficient screening for BC is an essential part of women’s healthcare [3]. The survival rate of BC varies among different parts of the world and is lower in low-income countries. This lower survival rate can be attributed to a lack of awareness and early detection programs, which results in a high proportion of women presenting with advanced disease. Since BC mortality is therefore related to the sensitivity of tumor detection methods used, the development of new detection methods capable of early tumor identification has been a highly active area of research for several decades [7,8]. Ideally, new schemes should be non-invasive, simple in both usage and result analysis, and inexpensive for implementation. Currently, the main approach for the early detection of BC is by screening mammography, which has been proven to reduce BC mortality. However, one of the limitations of screening mammography is the ability to detect small tumors in dense breast tissue [9]. The overall sensitivity of mammography is 75–85% but decreases to 30–50% in dense breast tissue [10]. One of the new methods that can overcome this limitation is dual-energy digital mammography [11,12]. This approach consists of high- and low-energy digital mammograms following the administration of an iodine-based contrast agent. However, the improved resolution of this method is achieved by the exposure of the breast to an increased dose of X-ray irradiation and contrast material injection. An additional method is magnetic resonance imaging (MRI) imaging [13]. MRI imaging has become increasingly important in the detection and delineation of BC in daily practice. MRI is highly sensitive in dense breasts, where mammography sensitivity is low. However, the major drawback of the MRI imaging technique is its high cost. Another method, based on identifying differences in electric properties between normal breast tissue and carcinoma [14], is electrical impedance tomography (EIT) [15]. This imaging method maps the body’s conductivity from superficial skin current measurements and has been shown to have a good ability to identify tumor abnormalities. Another pathway towards BC detection, which overcomes limits such as dense breast tissue and high cost, is through exhaled breath analysis [8,16,17,18,19,20,21]. It is well established that exhaled breath contains volatile organic compounds (VOCs) produced by biochemical reactions that occur in the human body. Consequently, the compositional analysis of exhaled breath can supply information regarding our medical condition [22]. Various illnesses have been identified by biomarkers in exhaled breath (EB), including chronic obstructive pulmonary disease [23], pulmonary diseases [24], diabetes [25], lung cancer [26], and pancreatic cancer [27]. As a result, exhaled breath analysis has become a very active research and diagnostic field during the last two decades [16,17,18,19,20,21,28,29,30,31,32,33,34,35,36,37]. The prosperity of EB analysis-based research is partially due to the development of small, easy-to-use, and inexpensive detectors, also known as electronic noses (ENs) [38,39,40,41,42]. Similar to the olfactory system, an EN uses an array of non-specific sensors. The EN does not provide precise information on the chemical composition of the exhaled breath gas mixture but a pattern of sensor signals that represent the EB composition. The EN data are usually analyzed using machine learning techniques [43,44,45,46]. These include different algorithms to identify patterns in the data and use them for data classification, allowing efficient predictions on such data.

In this work, exhaled breath collected from 267 women is measured using the commercially available Cyranose 320 EN. The measurement results then undergo feature extraction and are classified using an SVM model. The developed method exhibits accuracy, precision, and specificity values of 91.0%.

## 2. Experimental and Computational Methods

### 2.1. Exhaled Breath Collection

All samples were collected in the Breast Health Center in Soroka Medical Center, Beer Sheva. Exhaled breath was collected using Teldar 1 L sample collection bags (Cel Scientific Corp. Cerritos, CA, USA). Patients exhaled into a plastic tube (4 mm inner diameter) connected to the sampling bag, filling it to about 90% of the bag’s volume. For measurements, 20 mL of a sample was withdrawn from the sample bag to a disposable plastic syringe through the septa located on the Tedlar bag. Samples were measured within 4 h of sampling.

### 2.2. Electronic Nose

Exhale breath analysis was performed using the Cyranose 320 (Sensigent Intelligent Sensing Solutions, Baldwin Park, CA, USA). This EN has 32 polymer-based sensors, each with different sensitivity to various gases. Measurements were carried out in three steps. First, ambient air would flow through the EN for 25 s. This step serves as the baseline purge. Next, the sample in the syringe would flow into the EN for 40 s. Finally, the syringe was disconnected, and ambient air was drawn into the EN until the measurement was completed. The total measurement time was two minutes. The electronic nose output signals for each sample were recorded and saved. Of the EN’s 32 sensors, four showed sensitivity towards humidity and were therefore not used in this work.

### 2.3. Subjects

The sick women’s samples were taken from patients who were diagnosed as having breast cancer based on physical or mammography tests prior to any surgery. Sick women were identified as having breast cancer by biopsy tests. The control group consisted of healthy women who did not present any kind of cancer, pregnancy, or acute inflammation at the time of sample collection.

### 2.4. Data Analysis Method

Support vector machine (SVM) [47,48] was used for data classification. Hyperopt was used to obtain hyperparameters [46], and the parameters obtained were optimized with respect to the accuracy matrix using Monte Carlo cross-validation with 100 iterations, with each iteration containing 20% of the data, as the validation set.

## 3. Results

### 3.1. Exhaled Breath Collection

**Figure 1:**
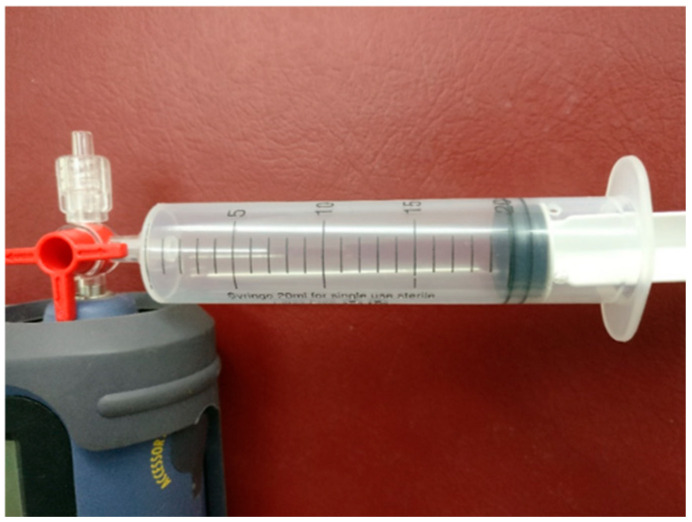
Injection of a sample into the EN using a disposable syringe.

Direct exhaled breath data collection is very complex to analyze. The signal obtained by the EN may be influenced by the condition of the patient. The signal may be altered and exhibit very high noise levels depending on breathing intensity and rate. To overcome this, patients were instructed to breathe into a gas collection bag, as suggested by the European Respiratory Society guide [49]. The collection bag then served as a reservoir, allowing the injection of a well-defined volume of exhaled breath samples for all patients (Figure 1). Samples were kept in ambient conditions and measured within 4 h of sampling. Samples were collected from every patient who visited the physician and agreed to participate in the research, without any limitations or requirements from the patient.

### 3.2. Exhaled Breath Data Processing

It has been previously shown that the EN sensor response data require some degree of processing prior to their classification [36]. Before processing, unnecessary information (response from unused sensors, instrument log information) was deleted. The first step of the data processing consisted of extraction of the effective signal duration and its normalization since only a fraction of the total measurement time is necessary for classification. For each sensor response, a moving difference window was used to detect the beginning and end of the main signal, and the rest of the measurement data were removed.

**Figure 2:**
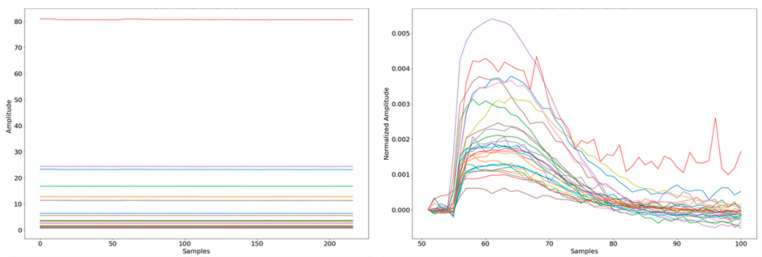
A typical sample prior to (**left**) and post (**right**)-normalization. Each line represents a different sensor. The results are presented after the extraction of the effective signal.

Next, outlier samples were manually removed from the data set. To accomplish this, each sensor value was normalized using Equation (1). A representative sample before and after normalization is displayed in Figure 2.

(1) $$S_{normalized}=\frac{S_{i}-S_{0}}{S_{0}}$$

where *S_i_* is the signal at time *i*, and *S*_0_ is the signal at time 0. It should be noted that the normalized results were only used for manual evaluation and not for classification. During the sample evaluation, 51 samples were found to contain no noticeable peaks (relative to a typical sample) and were removed from the data set. The criterion for sample removal was no signal higher than three standard variations of the background signal observed. The removed samples were all measured after more than 4 h from their measurement and had low signal intensities, which led to a low signal-to-noise ratio. Notably, none of the samples measured soon after (within 4 h) their collection were found to be noisy, indicating the importance of rapid sample measurement. Of the remaining 216 samples, 194 were randomly chosen for the model training set. The test set consisted of the remaining 22 samples. The final part of the data processing was applying a moving average filter to the data with a window size of three samples to filter out noises in the measurements.

### 3.3. Feature Extraction

Feature extraction is a process of finding the most relevant variables for a predictive model and is an important step in machine learning [47]. For feature extraction, the measurement data of each of the 28 sensors employed were encapsulated using 33 descriptive features. The features include basic statistics (maximum, minimum, amplitude, mean, median, standard deviation, interquartile range, kurtosis, and skewness), time-related features (time required for signal to rise from 10% to 90% of its maximum value and decrease back from 90% to 10%, time over mean, time over median, time over 90%, time from start to maximum, time of max slope, and time of min slope), area-related features (total area under the curve, area between start and max value, area over the mean, area over the median, and area over 90%), ratio features (area to amplitude ratio, area over 90% to time over 90%, area over mean to time over mean, area over median to time over median, and area start to max to time start to max), and slope-related features (maximum, minimum, mean, median, standard deviation, and interquartile range slopes). Each sample was, therefore, represented as a single line comprising 924 features (33 descriptive features for each of 28 sensors), which was then used for model selection.

### 3.4. Model Selection

Determining whether an exhaled breath sample belongs to a sick or healthy patient requires the use of a binary classification algorithm. SVM, specifically the C-Support Vector Classification (SVC) algorithm, was chosen for this task. This model was chosen as it is often useful when the number of parameters is high while the data set is not very large. It is possible that with a much larger database, a different model would be able to obtain superior results. The environment used to build the model was Python (version 3.10), and the library used was scikit-learn [50]. The kernel was a radial basis function kernel, or Gaussian kernel. This kernel is very versatile and efficient for capturing non-linear relationships.

Several steps were taken before further optimization of the model. First, all features with a variance of zero across all samples were removed (a total of 54 features). Of the remaining 870 features, the top 3% features (according to the ANOVA F-value) were selected, and the rest were discarded. Therefore, once all of the data processing was complete, each sample was represented by 27 features (detailed in the Supplementary Materials).

**Figure 3:**
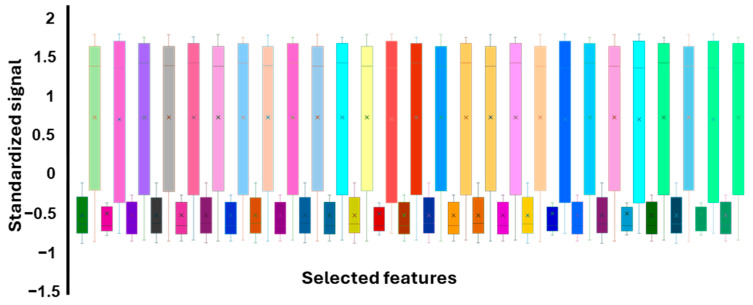
Boxchart of all standardized features selected for the model. Healthy subjects’ results are darker colored and can be seen to be more narrowly spread and negative than the sick subjects’ results. The sample legend is provided in the Supplementary Materials, X represents the mean of the dataset.

The selected features were then standardized, as SVM can be sensitive to changes in the order of magnitude in the data set. For this, each column in the data set was standardized by subtracting the mean and scaling to unit variance. A boxchart of the standardized selected features for sick and healthy subjects is presented in Figure 3. It can be observed that the results for the healthy subjects are narrower and more negative than the sick subjects’ results.

**Figure 4:**
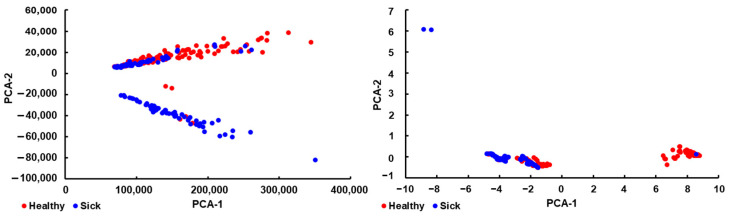
PCA plots for all features (**left**) and the selected, standardized features (**right**). Data from healthy subjects is in red, and from sick subjects in blue. Additional plots (PCA-3 and PCA-4) are presented in the Supplementary Materials.

PCA plots of the entire dataset (all features) and of the features selected for the model after standardization are presented in Figure 4 (with additional plots in the Supplementary Materials). The plots show the results obtained from both sick and healthy subjects. It can be observed that the standardized selected features display a better separation of the two sample types.

### 3.5. Model Optimization

The training data for the model were composed of 194 of the samples (80 sick, 114 healthy), and the test data were composed of 22 samples (11 sick, 11 healthy). The optimization process was performed using hyperopt [51], aiming to obtain hyper-parameters that yield the best performance for the SVM model. The SVM model was then optimized with respect to the accuracy matrix using a validation set by applying a Monte Carlo cross-validation with 100 iterations, with each iteration containing 20% of the data. In the optimization process, hyperopt runs the model using random parameters (from a provided range; see Supplementary Materials) and can yield a different model each time; however, on average, the obtained parameters make a model of ca. 90% accuracy, with considerably low variance compared to the train set size. The optimized parameters are selected, chosen since they achieved relatively small variance and bias scores for the train and validation sets. The chosen parameters for the model are presented in Table 1.

**Figure 5:**
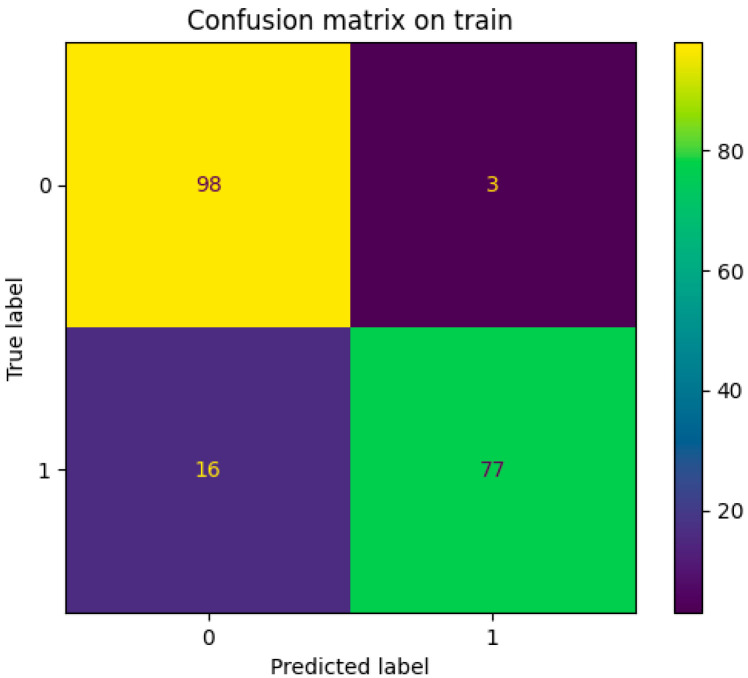
Confusion matrix for the train data set. Labels 0 and 1 represent healthy and sick subjects, correspondingly.

After receiving the optimized parameters, the tuned model was trained on all of the training samples, with scores for *recall*, *precision*, *accuracy*, and *specificity*. Recall represents the fraction of “true positive results” out of the total positive results (true positive and false negative). Precision represents the fraction of “true positive results” out of all positive results (true and false). Accuracy is the fraction of the true positive and true negative results out of the total number of positives and negatives. Specificity is the fraction of true negatives out of total negative results (true negative and false positive). The train and validation scores after parameter training are detailed in Table 2. The confusion matrix of the train data, used to visualize an algorithm’s performance, is presented in Figure 5.

### 3.6. Model Performance

**Figure 6:**
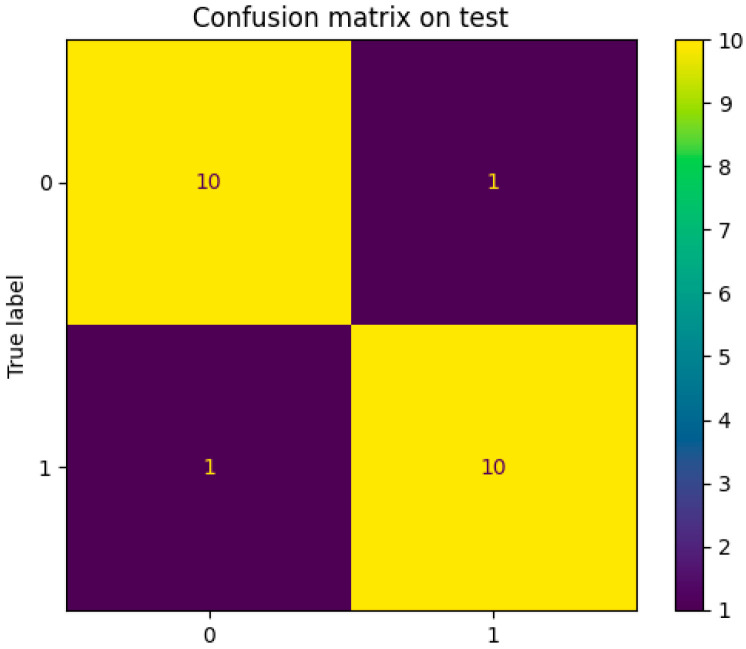
Confusion matrix for the test data set. Labels 0 and 1 represent healthy and sick subjects, correspondingly.

The performance of the model was assessed using the 22 samples of test data. The scores obtained using the model are presented in Table 3, and the confusion matrix is presented in Figure 6.

## 4. Discussion

This study used 242 exhaled breath samples to produce a model that allows for a facile diagnosis of breast cancer. Gas chromatography has previously been used for BC determination using exhaled breath, with fine results [18,19,20,52,53]. However, that technique is much more costly and requires more expertise relative to the EN. While novel ENs are constantly researched and produced, the use of a commercial EN makes the method readily available for application. In addition to previous work in our group [36], there have been several publications using the Cyranose 320 EN for breast cancer analysis [54,55]. Comparing these works with the research presented here, their main strength is the significantly higher number of samples obtained (443 and 899). This allowed them to examine and evaluate the effect of many factors, such as age, smoking, asthma, and diabetes, on the method. The smaller number of participants in our research limits our ability to perform such analysis in a statistically significant manner. In addition, a larger data pool, such as in the mentioned works, can be expected to improve the model optimization process and the final method parameters. Consequently, the correct prediction value obtained by Lorena Díaz de León-Martínez et al. was 98.7%, while Hsiao-Yu Yang et al. obtained a value of 97%, both higher than the 91% value obtained in this work. Future work building on the foundations presented here should look to significantly increase the number of participants in the study, hopefully obtaining well over a thousand samples. On the other hand, this work has several strengths. The main one is the simplicity and robustness of the method. Lorena Díaz de León-Martínez and colleagues required 5 h of fasting, no smoking, no oral hygiene, and no medication prior to exhaled breath collection. Hsiao-Yu Yang and colleagues required 8 h of fasting and collected alveolar air after anesthetic drugs were employed for surgery. While these limitations can improve the data quality from exhaled breath, especially the use of endotracheal intubation to obtain the purest alveolar air, our method placed no such limits on the participants of the research. As a result, samples in our data are not as “neat” and more varied. This should make our method more robust, allowing it to remain less affected by small variations [56]. Without any requirements from the patient, samples could be collected from anyone visiting the physician or even at the workplace, not necessitating the presence of a physician for BC screening. In addition to the method’s robustness, our measurement procedure is very simple, as it is performed at ambient temperature, and has a total measurement time of 2 min, making it quite rapid, and it requires no additional equipment other than the Cyranose 320. We have found that the collected breath samples were not very stable, with samples measured more than 4 h after collection exhibiting a high degree of noise. This might be improved by storing the samples in a cooler environment prior to measurement. However, the simplicity and short measurement time of the method make the simplest solution to perform the measurement shortly after sample collection. Finally, we presented our feature extraction process in a detailed manner, which could be useful for future researchers. Previous works extracted features using software that provides few details [54] or omitted feature extraction from their analysis process [55].

Currently, ENs are unlikely to be able to replace very established detection methods such as mammography, MRI, and biopsies. However, the simplicity and low cost of analysis make BC screening by EN a very promising option for developing countries, wherein early detection could significantly lower the BC mortality rate. In addition, the non-invasive nature of the technique makes it simple to implement in many clinics. As the pool of exhaled breath data increases, the use of AI big data analysis [57] should further improve classification models and increase the number of diseases recognized by exhaled breath.

## 5. Conclusions

This study aimed to develop a robust, accurate, and highly reliable screening method for the early detection of BC in routine screening using exhaled breath (EB) analysis. Method advantages include its low cost, simplicity, and being non-invasive. Samples were collected from 242 women (120 cancer patients, 122 healthy) and analyzed using a commercial EN. The EN is composed of 32 polymer-based sensors that change their electrical conductivity when exposed to gas mixtures. Feature extraction was applied to the results to minimize the data obtained to their most relevant features. An SVM model was adopted for data classification. After training the model, the validation set results were 80, 94, 88, and 95% for recall, precision, accuracy, and specificity, correspondingly. The analysis of 22 unknown samples gave results of 91% in accuracy, specificity, recall, and precision.

This study demonstrates the potential of EN as a non-invasive, simple, safe, painless, and inexpensive diagnosis method and demonstrates the importance of classification models after features have been extracted from the data. Further research with larger sets of data, larger sample volumes, and optimized sensor arrays could further improve the results obtained in this work.

## Figures and Tables

**Table 1:** The optimized parameters obtained for the SVM model.

Parameter	Value
C	66
Coef0	66
Degree	10
Gamma	2.5
Tol	0.02

**Table 2:** The obtained scores for the train and validation sets.

Parameter	Train	Validation
Recall	0.80 ± 0.02	0.80 ± 0.11
Precision	0.95 ± 0.01	0.94 ± 0.06
Accuracy	0.89 ± 0.01	0.88 ± 0.06
Specificity	0.96 ± 0.01	0.95 ± 0.05

**Table 3:** The obtained scores for the test set.

Parameter	Test
Recall	0.91
Precision	0.91
Accuracy	0.91
Specificity	0.91

## Data Availability

The original contributions presented in this study are included in the article/Supplementary Materials. Further inquiries can be directed to the corresponding author(s).

## Supplementary Materials

The following supporting information can be downloaded at: https://www.mdpi.com/article/10.3390/s25072210/s1, Table S1: Selected features for classification model, Figure S1: Boxchart of all standardized features selected for the model, with legend, Figure S2: PCA plots (PCA-3 and PCA-4) for all features and the selected, standardized features, Table S2: Parameter range used in model optimization.

## Abbreviations

The following abbreviations are used in this manuscript:

BC	breast cancer
VOCs	volatile organic compounds
EB	exhaled breath
EN	electronic nose
SVM	support vector machine
