# Supplementary material for: Chemical Nose-Based Non-Invasive Detection of Breast Cancer Using Exhaled Breath
Source: Sensors (Basel). 2025 Mar 31;25(7):2210. doi: 10.3390/s25072210 (PMC11991366; doi:10.3390/s25072210)
Supplement: Supplementary file 1 [file sensors-25-02210-s001.zip › sensors-3530849-supplementary.pdf]

Chemical nose based non-invasive detection of breast cancer using exhaled breath

Yosef Matana[1], Shai Libson[2], Barak Amihood,[3] Zvi Boger[1,3], David Lieberman[4], Offer Zeiri[5*], Yehuda Zeiri[1*]

[1] Biomedical Engineering, Ben-Gurion University of the Negev, Israel

[2] Breast Health Center Soroka Medical Center, Ben-Gurion University, Israel

[3] OPTIMAL – Industrial Neural Systems, Israel

[4] Pulmonary Unit, Soroka University Medical Center and the Faculty of Health Sciences, Ben-Gurion University of the Negev, Israel

[5] Department of Analytical Chemistry, Nuclear Research Center Negev, Israel

Selected features

Of 870 features obtained, the top 3% were chosen for sample classification. The chosen features are displayed in table S1.

Table S1. Selected features for classification model

Sensor number	Feature
9	Maximum value
15	Maximum value
19	Maximum value
9	Minimum value
19	Minimum value
9	Mean value
19	Mean value
9	Median value
19	Median value
9	Total area
19	Total area
9	Area over median
15	Area over median
19	Area over median
9	Area over 90%
19	Area over 90%
9	area over 90% to time over 90%
19	area over 90% to time over 90%
9	area over mean to time over mean
15	area over mean to time over mean
19	area over mean to time over mean
9	area over median to time over median

15	area over median to time over median
19	area over median to time over median
9	area start to max to time start to max
15	area start to max to time start to max
19	area start to max to time start to max

Boxchart plot legend

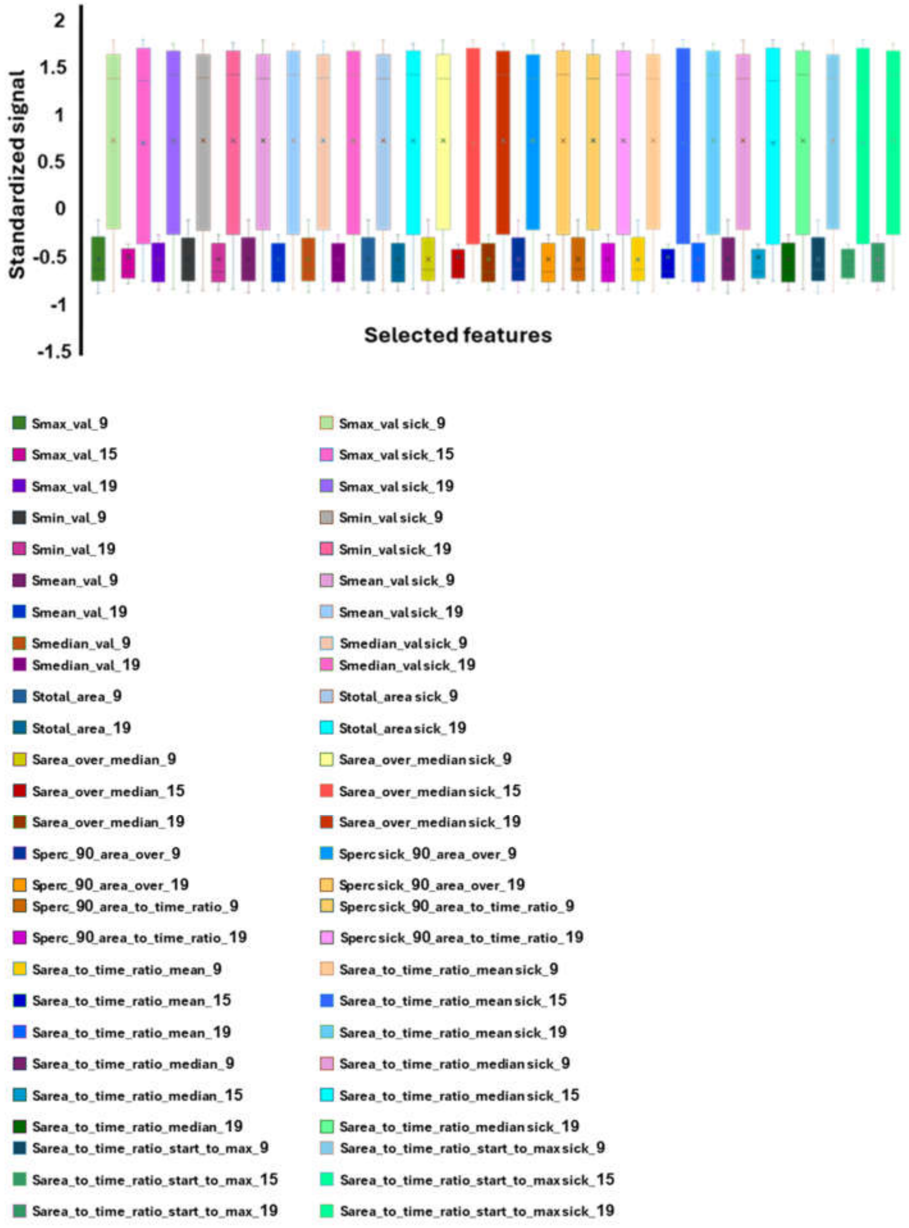

Figure S1: Boxchart of all standardized features selected for the model, with legend. Healthy subjects' results are darker colored and can be seen to be more narrowly spread and negative than the sick subjects' results.

Additional PCA plots

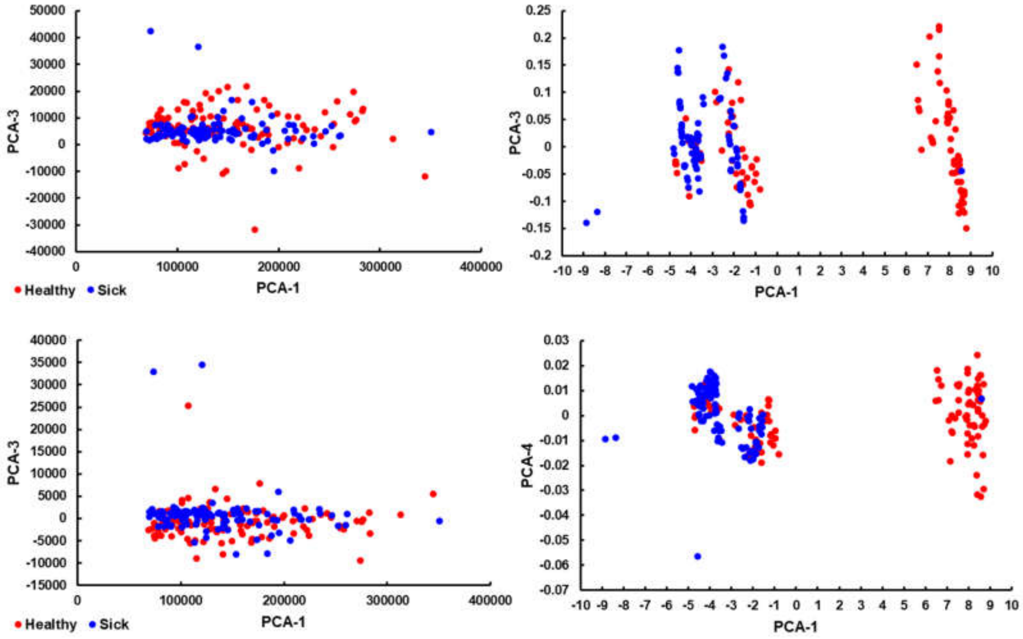

Figure S2: PCA plots (PCA-3 and PCA-4) for all features (left) and the selected, standardized features (right). Data from healthy subjects is in red, and from sick subjects in blue.

Model parameter range

The optimization process is performed by running the model using random parameters within a chosen range. The model parameters resulting in the best variance and bias scores for the train and validation sets was then chosen for the model. The range of the random parameters, chosen using Hyperopt, is displayed in table S2.

Table S2. Parameter range used in model optimization

Parameter	range
**C**	exp(-5) to exp(5)
**Coef0**	exp(-5) to exp(5)
**Degree**	exp(2) to exp(100)
**Gamma**	exp(-5) to exp(5)
**Tol**	exp(-6) to exp(-1)
